# Economic evaluation of the prevention of falls resulting from missed care in polish hospitals

**DOI:** 10.3389/fpubh.2024.1228471

**Published:** 2024-09-16

**Authors:** Beata Wieczorek-Wójcik, Aleksandra Gaworska-Krzemińska, Aleksander Jerzy Owczarek, Dorota Kilańska

**Affiliations:** ^1^Department of Nursing and Medical Rescue, Pomeranian University, Słupsk, Poland; ^2^Institute of Nursing and Midwifery, Medical University of Gdańsk, Gdańsk, Poland; ^3^Health Promotion and Obesity Management Unit, Department of Pathophysiology, Faculty of Medical Sciences, Medical University of Silesia, Katowice, Poland; ^4^Department of Coordinated Care, Medical University of Lodz, Łódź, Poland

**Keywords:** cost-effectiveness analysis, cost-benefit analysis, accountable care organization, falls, patient outcome assessment, patient safety, nursing staff

## Abstract

**Objectives:**

Falls are associated with increased morbidity, mortality, prolonged hospitalization and an increase in the cost of treatment in hospitals. They contribute to the deterioration of fitness and quality of life, especially among older patients, thus posing a serious social and economic problem. They increase the risk of premature death. Falls are adverse, costly, and potentially preventable. The aim of the study was to analyze the cost-effectiveness of avoiding one fall by nurse care provided by the nurses with higher education, from the perspective of the health service provider.

**Methods:**

The economic analysis included and compared only the cost of nurse intervention measured by the hours of care provided with higher education in non-surgical departments (40.5%) with higher time spend by nurses with higher education level an increase in the number of hours by 10% (50.5%) to avoid one fall. The time horizon for the study is 1 year (2021). Cost-effectiveness and Cost–benefit analysis were performed. All registered falls of all hospitalized patients were included in the study.

**Results:**

In the analyzed was based on the case control study where, 7,305 patients were hospitalized, which amounted to 41,762 patient care days. Care was provided by 100 nurses, including 40 nurses with bachelor’s degrees and nurses with Master of Science in Nursing. Increasing the hours number of high-educated nurses care by 10% in non-surgical departments decreased the chance for falls by 9%; however, this dependence was statistically insignificant (OR = 1.09; 95% CI: 0.72–1.65; *p* = 0.65). After the intervention (a 10% increase in Bachelor’s Degrees/Master of Science in Nursing hours), the number of additional Bachelor’s Degrees/Master of Science hours was 6100.5, and the cost was USD 7630.4. The intervention eliminated four falls. The cost of preventing one fall is CER = USD 1697.1.

**Conclusion:**

The results of these studies broaden the understanding of the relationship among nursing education, falls, and the economic outcomes of hospital care. According to the authors, the proposed intervention has an economic justification.

## 1 Introduction

“Delivering high value for patients must be the central and general goal of every health care organization” (1). The European Commission has consistently pursued the Value-based Health Care (VBHC) policy by recommending the extension of the procurement criteria for selecting the providers of health services equal to or exceeding EUR 750,000, and adding the criterion of “best value for money” (2). The value of nursing care for the patient, the healthcare system, and the payer has been proven in many studies, demonstrating not only the costs of insufficient care but also the benefits of adequate nursing staffing (3–17). Adequate staffing and access to necessary resources are vital for nurses to deliver high-quality care, which is conducive to reducing long-term costs associated with poor patient outcomes. Additionally, allowing nurses to work to their full scopes of practice prevents the waste of valuable human capital, particularly in a profession dominated by women, where their skills are often underused (18).

Value-based Health Care is becoming a priority for healthcare management (19). Productivity and sustainability are essential parts of VBHC, where patient experience and safety are quality indicators. Suitable Development Goals (SDGs) include “Good Health and Well-Being.” In order to attain the Good Health goal, it is essential to provide the extent of nursing care that is necessary to achieve a positive health outcome. Achieving positive health outcome is possible through nursing services that contribute to shortening the length of hospital stay and improving the patient’s health status, thus adding health to years (20). Over the past 30 years, the relationship between structural nursing-sensitive indicators and patient outcomes (e.g., falls or lack thereof) has been repeatedly proven (21–23). However, few studies demonstrate the value of nursing care at the process level in influencing health outcomes (21, 22, 24). Delivering high-quality care means providing appropriate care to the right patient, in the right way, at the right time (25), in safe and comfortable working conditions, with positive treatment outcomes achieved for patients (26). The quality of nursing care (27) and patient safety (28, 29) are influenced by workload, which may be affected by a lack of resources. Studies have confirmed that the current minimum nurse staffing standards do not guarantee the desired level of quality in medical services or the implementation of accepted care standards (30). It has also been proven that having an adequate number of nurses is not enough; their qualifications are also crucial for ensuring good quality care (25, 28, 31). Employing better educated nurses appears to make a substantial positive impact on patient outcomes, patient experience, and hospital costs (32). The consequences of the fall include not only pain and suffering (patient perspective), but also translate to prolonged hospital stay (from the hospital’s perspective), and consequently, high costs of treatment and care and increased demand for care (from the payer’s perspective) (33–36). Falls are associated with increased morbidity, mortality (patient perspective), and prolonged hospitalization (patient, payer, and hospital perspective) (37) and an increase in the cost of treatment in hospitals (payer perspective) (16). They contribute to the deterioration of fitness and quality of life (patient perspective) (38), especially among older patients (39) thus posing a serious social and economic problem (40). They increase the risk of premature death (40). In Polish hospitals, the most common adverse events related to nursing care include nosocomial infections, pressure ulcers, falls, and incorrect assessment of the patient’s condition or failure to detect symptoms signaling a deteriorating condition while conducting the assessment of the patient’s condition (41). The costs of preventing falls have been analyzed, and factors that can eliminate them are known; they include improving the quality of care by ensuring an adequate number of nurses to meet patients’ needs (42). Such data are not available in Poland. Moreover, unlike in other countries, hospitals in Poland do not maintain fall registries (43). Additionally, it should be emphasized that the standard practice is to meet the minimum staffing norms for nurses, which do not correspond to quality care indicators. The relationship between staffing levels, qualifications, and falls is not known. A review of the literature did not reveal any publications analyzing these variables, and cost analyses are also lacking (44).

There is a link between higher education of nurses (Bachelor’s Degrees—BSN/Master of Science—MScN) with a reduction in fall rate (45). Many international studies have also confirmed the link between nurse qualifications and: (a) patient outcomes (mortality and satisfaction)—24 studies; (b) knowledge and skills, commitment and job satisfaction, status improvement, and self-confidence—54 studies; (c) organizational benefits including staff stability, health care perception, and care costs—six studies (46). It has been proven that the higher the number of nurses with a BSc or MScN degree, the lower the fall rate per 1,000 patient-days (47). Additionally, a higher Nursing Hours per Patient Day (NHPPD) provided by nurses to severely ill patients is associated with a lower fall rate and more accurate assessments of the patient’s health status (12). Better education and training of nurses in surgical and non-surgical departments are essential in meeting customer expectations and maintaining patient care outcomes (48), and they are critical in achieving value for the patient.

National Health Fund do not indicate the proportion of nurses with higher education to other nursing staff. Training programs for nurses focus on increasing self-confidence, knowledge, critical thinking skills and improving interpersonal skills. Unfortunately, nursing leaders have yet to shape the quality of nursing care based on services selected for their quality-to-price ratio.

The current direction of change in Poland is also slowly toward paying for treatment results rather than procedures (49). As nursing leaders, we see that missed nursing care is endemic across all sectors, and falls are a proven consequence of that issue (50). During the hospital care, falls affect patient mobility and mean more staff involvement in care delivery (38) also due to pressure ulcers, new infections and patient mortality (51, 52).

Falls are among the most common adverse events associated with nursing care. Falls are one of the indicator of the nursing indicators sensitive to nursing care (e.g., C-HOBIC) (6, 53) and recognized in nursing quality indicator systems, such as Nursing Quality Indicators for Reporting and Evaluation® (NQuIRE®) (54). Only a few nursing-sensitive indicators are recognized and used worldwide; they are measured at the structural, process, or outcome levels (22). However, most existing indicators are measured at the structural level (e.g., nursing staff) (55) or at the outcome level (e.g., mortality and adverse events such as urinary tract infections and pressure ulcers) (55–59). In contrast, nursing-sensitive indicators at the process level (e.g., mobility—falls, nutritional support, and vital sign measurements) are rarely used routinely (22, 60), although the failure to meet nursing-sensitive process indicators, sometimes referred to as missed nursing care (50, 61) or nursing care neglect (60), poses a serious problem in healthcare (62, 63). Databases play an important role in ensuring high and consistent quality of healthcare by continuously monitoring treatment, care, and rehabilitation for clinical quality (32, 64). They are increasingly used in healthcare planning and in setting priorities to improve patient outcomes. However, there is often a lack of knowledge about effective reporting and measurement of nursing activities (65). The Quality Act, adopted in Poland in 2023 for the first time in the history of healthcare (66) does not include Nursing Sensitive Indicators including recorded falls despite their proven significance (67).

According to published data, fall incidence rates range from 1.3 to 8.9 per 1,000 patient days (15, 17, 68). There was some tendency for units in larger hospitals to have lower unassisted fall rates. For medical–surgical units, the estimated average fall rate was 10% (95% CI: 5–16%) higher in teaching hospitals (17). Falls are among the main quality of care indicators that are sensitive to nursing care (47). Many of them can be prevented through proper and safe care (69). Healthcare spending in Poland is among the lowest in the EU (70), resulting in prioritizing the low value of services and saving on nursing care. In Poland, nursing care is generally considered a cost. Studies have shown that nurse care costs account for only 25.5–30.1% of the yearly hospital budget (71). Increasing the number of nurses in the healthcare system has impact on the Gross Domestic Product (GDP) growth—the magnitude of the effect of nurse staffing on GDP is 1.52 in Poland, which is among the highest in Europe (72). The procurement criteria of the Polish (19). This paper presents a health technology assessment (HTA) to determine the value of BSN/MScN care.

The aim of the study was to analyze the cost-effectiveness of avoiding one fall by increasing the percentage of nurses with higher education.

## 2 Materials and methods

The clinical assessment of the impact of BSN/MScN care in medical departments on the incidence of falls was carried out in a case–control (retrospective) study. The analysis concerned patients hospitalized in the period from January 1, 2021 to December 31, 2021 in the following departments: cardiology, neurology, internal disease, and pulmonary diseases, who suffered a fall.

The study site was the Specialist Hospital in Wejherowo [Szpital Specjalistyczny w Wejherowie], one of the four Pomeranian Hospitals in the Pomeranian Region. The hospital provides specialized care at the third reference level of the hospital network for 20% of the region’s population, offering several unique services and certain specialized medical facilities, including specialized cardiac surgery, cardiology, neurology, and orthopedic procedures, as well as an Emergency Department (ED) and intensive care. In 2021, 34,167 patients were hospitalized (90,853 patient-days) in seven medical wards, and 12,472 surgeries were performed, accounting for 21% of the hospital’s patients, with 55% of patients admitted in the urgent (unplanned) mode through the ED.

### 2.1 Study design and setting

Cost-effectiveness Analysis (CEA) and Cost Benefit Analysis (CBA) was evaluated based on the clinical case study. The analysis scopes were established based on the following: assumptions:

- population—patients (women and men) over 18 years of age, hospitalized in internal disease, cardiology, neurology, and pulmonary disease during a 12-month period;
- intervention: a 10% increase in the number of hours of care provided by BSN/MScN nurses up to 50.5%;
- comparator: current working hours of nurses with higher education (40.5%);
- outcome: all fall patients, regardless of whether they were injured during the fall; patients and direct cost per care per prevented fall (CER).

The study compared the cost of the current number of hours of care provided by nurses with higher education in non-surgical departments (40.5%) and an increase in the number of her hours by 10% (50.5%) to avoid one fall (the perspective of the provider).

To calculate the cost of avoiding one fall (from the hospital’s perspective), the cost of increasing the proportion of BSN/MScN nurses by 10% was compared with the current standard level of care. The comparison between standard care and the 10% BSN/MScN intervention was based on previous analyses conducted at the studied hospital, which indicated a relationship between the percentage of BSN/MScN nurses and falls in non-surgical wards, when the proportion of BSN/MScN is increased by 10%, and it was arbitrarily determined that the 10% intervention would be used for the calculations in this study. The inclusion criterion for the non-surgical wards in the study was based on the fact that there was no demonstrated relationship between NHPPD and higher education levels in surgical wards (73). The time horizon for the study is 1 year (2021). The study was designed according to the CHEERS protocol (74).

### 2.2 Collection of data

The data used in the analysis comes from actual calculations and hospital data. The parameters used in the analysis include (1) the actual number of patient and patient days in 2021 (number of patient days is the number of patients admitted to hospital multiplied by the number of days spent in hospital), for which we calculated fall incidence per 1,000 patient days; The study included patients from four non-surgical departments, including 7,305 patients who were hospitalized, which constituted 41,762 patient days. (2) the number of hours provided by nurses, including nurses with higher education, which was monitored daily and recorded in Excel; (3) the average cost of an hour of work performed by nurses with secondary and higher education as of 2021; and (4) the number of falls. The definition of a fall was: an event that resulted in a person unintentionally landing on the ground, floor or other lower level (75). Data on falls were collected in Electronical Health Record (EHR)—EPR: CLININET. Upon admission to the hospital, each patient had his or her risk of falls assessed according to the MORSE scale (used since 2012). The variables assessed were summed, then the system calculated a risk score for each patient. The variables assessed (five risk factors) were: history of falls, multi-morbidities, mobility support, mobility assessment, mental state. The falls register is maintained by the falls team, using a daily reporting system. The analysis of falls concerned monthly and annual periods; the incidence of falls was calculated per 1,000 person-days in the analyzed departments.

### 2.3 Data analysis

The CEA and CBA analysis is based on the assumption that patients will receive additional hours of care provided by nurses with a BSN/MScN title. The cost of nurses’ salaries was calculated in PLN, and then converted into USD according to the average National Bank of Poland (NBP) exchange rate from 2021 (4.1051). The cost of 1 h of care provided by a nurse with a BSN/MScN degree was calculated by dividing the total salary of nurses with higher education by the actual number of hours of nurses with a BSN/MScN degree. The cost of 1 h of work of a general nurse (i.e., without higher education) was calculated analogically. The intervention involved increasing the number of hours of nurses with higher education, which means an increase in the level of education, and not the employment of nurses, so we calculated the incremental cost (other costs will still be valid after the intervention).

The cost of a BSN/MScN nurse’s working hour was calculated according to the Cookson methodology, i.e., by subtracting the cost of an hour of a nurse without a higher education from the cost of an hour of a graduate nurse. Other costs of care were assumed to be non-differentiating.

In the cost-effectiveness analysis, two measures of health effect were adopted: (1) a fall that can be prevented; (2) the falls that occurred during hospitalization were monitored daily and recorded in Excel. The next step involved calculating the number of falls that could be avoided by increasing the number of BSN/MScN nurses’ working hours by 10%. The result was obtained by dividing the cost of additional hours of BSN/MScN nurses by the number of avoidable falls. To calculate the number of avoidable falls we used the coefficient from previous study. This study showed correlation between education level and falls (backward stepwise regression result). However, there was no relationship between the number of Nursing Hours per Patient Day (NHPPD) and the severity of patients’ condition, and the incidence of falls (73). It was proved the relationship between the organizational change consisting in increasing the number of nurses with higher education by 10% and adverse events in hospitalized patients: deaths—971; rehospitalizations—2,129; bedsores—334; falls—198; pneumonia—143; urinary tract infections—159; and re-operations—153 and hospitalization time. This study included 44,809 patients. The more patients in Cat. III according to PCS, the more frequent the occurrence of falls in surgical departments. An increase in the percentage of patients in PCS category III by 10% = an increase in the incidence of falls by 0.158 per 1,000 patient days [0.0158 (0.0044) *p* < 0.01]; the more nurses with higher education, the less frequent the occurrence of falls in medical departments. A 10% increase in the percentage of nurses reduces the incidence of falls by 0.737 per 1,000 patient days [−0.0737 (0.0285) *p* < 0.05]; the more nurses with higher education, the fewer rehospitalizations in surgical and medical departments. An increase in the percentage of nurses by 10% causes a decrease in the incidence of rehospitalization by 24.74 per 1,000 patient days [−2.474 (0.570) *p* < 0.01] and 8.79 per 1,000 patient days [−0.879 (0.283) *p* < 0.05]; the more nurses with higher education, the fewer deaths in surgical and medical departments. An increase in the percentage of nurses by 10% causes a decrease in mortality by 3.25 per 1,000 patient days [−0.3250 (0.0448) *p* < 0.001] and 7.53 per 1,000 patient days [−0.7529 (0.1385) *p* < 0.001]. Moreover, in the case of medical departments, the mortality rate decreases with an increase in the nursing care rate [−6.8171 (2.3547) *p* < 0.05] (73). The cost of obtaining a unit of effect which is the cost of avoiding one fall (CER—The cost-effectiveness ratio) refers to the ratio of the cost of the intervention consisting in increasing the number of hours of BSN/MScN nurses by 10% to the health benefit of avoiding a fall was calculated by dividing the cost of additional hours of nurses with higher education by, respectively, the number of avoidable falls.

A cost–benefit analysis (CBA) was conducted in order to verify the cost-effectiveness threshold of the 10% increase in the number of hours of care provided by nurses with higher education and fall prevention from the perspective of the hospital. The number of falls avoidable by increasing the hours of nurses with higher education in non-surgical departments by 10% was the benefit, which was the benefit calculated for the analyzed departments. The next step was to establish the monetary benefit of avoidable falls by calculating the estimated value of nurses’ salaries (including projected growth) according to data from the available literature (25.5%) (76). This value was applied to the hospital budget calculated on the basis of the cost per day of hospitalization according to the hospital price list and then converted into avoidable falls. The CBA analysis was used to verify the cost-effectiveness of increasing the number of hours of care provided by BSN/MScN nurses. The gross monetary benefit was calculated by multiplying the percentage of nurses’ salaries in the hospital budget by the number of avoidable falls. We calculated how much needs be to spent in zloty (1 PLN ≈ 0.243 USD) to save 1 PLN. This analysis was carried out using cost–benefit ratio (CBR) and cost–benefit ratio (BCR). The result obtained in PLN was converted into USD.

GDP *per capita* was 37,800 USD in 2021 in Poland. The calculations aimed to determine how much we need to spend in zloty (1 PLN ≈ 0.243 USD) to save 1 PLN. The result obtained in PLN was converted into USD.

The study was approved by the Bioethics Committee of the Medical University of Gdansk under approval no. NKBBN/573/2022.

## 3 Results

In the analyzed period, 7,305 patients were hospitalized, which amounted to 41,762 patient days. The total number of beds in the analyzed non-surgical departments, i.e., pulmonary diseases, internal diseases, neurology, and cardiology, was 158. The average bed occupancy was 85.5%, and the average hospitalization time was 4.8 days. The mean cost of hospitalization based on the price list was USD 2,309 per patient (77).

Care was provided by 100 nurses, including 40 BSN/MScN nurses. The total number of hours of nursing care was 151,286 h, and the number of BSN/MScN nurse hours was 61004.9 (40.5%) before the intervention. After the intervention, the number of BSN/MScN nurse hours was 67105.4. The cost of BNS/MNS nurses’ hours was USD 548611.3 before and USD 556241.7 after the intervention. The difference in total salaries was USD 7907.8.

### 3.1 Participants’ characteristics

In the analyzed period 7,305 patients were hospitalized, which amounted to 41,762 patient days. In the study population, the number of women was—3117 (42.7%)—and the number of men was 4,188 (57.3%). The average age of patients in the general population was 64.2 years, including women—66.2 and men—63.2. The patient population who suffered a fall (64 patients) included 25 women (39%) and 39 men (61%). Main diagnosis according to ICD-10: Cancer—9 (14.1%);^1^ Stroke—11 (17.2%);^2^ Sepsis—6 (9.4%);^3^ Other disease nervous systems—8 (12.5%);^4^ Other respiratory diseases—5 (7.8%);^5^ Heart failure (N17.9)—2 (3.1%); Heart attack—3 (4.7%);^6^ Heart failure—2 (3.1%); COVID 19–3 (4.7%) (U07.1); Gastrointestinal disease—5 (7.8%);^7^ Kidney failure (N17.9)—4 (6.2%). The profile of patients who suffered a fall (including those who fell) in the analyzed period was compared according to ICD-10 and 80% of the most common causes of hospitalization were identified. A description of the clinical characteristics of the entire population is provided in Appendix to the article (Table 1).

**Table 1:** A characteristics of nurses included in the CEA analysis.

Characteristics of the population included in the CEA analysis													
	Characteristics of patients relative to costs						Characteristics of nurses relative to costs						
Departments	No patients	No. of PD	NB	BD	T Hospitalization	Hospitalization cost per patient (USD) as per hospital price list	No. of nurses in total	No. of BSN/MScN	No. of H RN	No. of H BSN/MScN before IC	No of BNS/MScN H after IC	Cost of BNS/MScN H before IC	Cost of BNS/MScN H after IC
Pulmonary diseases	970	7,817	26	90.6	4.8	1753.9	15	6	28149.0	11259.6	12,386	98830.8	101083.7
Internal diseases	2,062	13,195	45	95.6	6.4	2338.6	34	12	41614.0	14687.3	16,156	152565.9	154412.0
Neurology	1,268	9,388	36	82.6	5.2	2280.1	24	11	36228.0	16604.5	18,265	146204.8	148095.5
Cardiology	3,005	11,362	32	73.1	2.8	2114.4	27	11	45295.0	18453.5	20,299	151009.8	153171.1
Total non-surgical units	7,305	41,762	158	85.5	4.8	2309.3	100	40	151286.0	61004.9	67,105	548611.3	556241.7

BD, Bed occupation; H, hours; IC, Intervention; NB, No. of beds; NH, Nursing hours; BNS, Bachelor of Nursing Science; MScN, Master of Nursing Science; PD, Patient day; RN, Registered nurse; T, Time.

### 3.2 Falls analysis

In the analyzed non-surgical departments, there were 64 falls in 2021. An increase in the percentage of BSN/MScN nurses from 40.5 to 50.5% in non-surgical departments decreased the incidence of falls by 9%; however, this dependence was statistically insignificant (OR = 1.09; 95% CI: 0.72–1.65; *p* = 0.65). The most significant number of falls was recorded in the internal diseases department, while the cardiology department had the smallest one (73). As a result of the 10% increase in the working hours of BSN/MScN nurses, the number of falls decreased by 1 in each of the analyzed departments. The pre-intervention incidence of falls was 1.5 (BSN/MScN ratio = 40.5%), and the post-intervention rate was 1.4 (BSN/MScN ratio = 50.5%) per 1,000 patient days. The incremental change is 0.1 (Table 2).

**Table 2:** Clinical effect of the intervention.

Interventions and clinical effect before and after the intervention in relation to department structure in 2021						
Departments	Number of falls before intervention	Number of falls after intervention	Percentage of BSN/MScN nurses before intervention	Percentage of BSN/MScN nurses after intervention	Rate falls per 1,000 patient days before intervention	Rate falls per 1,000 patient days after intervention
Pulmonary diseases	11	10	40.0	50.0	1.1	1.0
Internal diseases	30	29	35.3	45.3	2.3	2.2
Neurology	13	12	45.8	55.8	1.4	1.3
Cardiology	10	9	40.7	50.7	0.9	0.8
Total non-surgical units	64	60	40.5	50.5	1.5	1.4

BNS, Bachelor of Nursing Science; MScN, Master of Nursing Science.

### 3.3 Intervention effects

After the intervention (a 10% increase in BSN/MScN hours), the number of additional BSN/MScN hours was 6100.5, and the cost was USD 7630.4. In Poland, the cost-effectiveness of technology is determined at the level of three times the value of GDP, which was 166,758 in 2021 (78). The intervention eliminated four falls. The cost of preventing one fall CER = USD 1697.1 (Table 3).

**Table 3:** Cost-effect ratio (CER).

Departments	Number of additional BSN/MScN nursing hours (10%)	Cost of additional graduate nursing hours (USD)	The Number of falls that are preventable through a 10% increase in BSN/MSN Nurses	Cost-effect ratio of avoiding one fall by increasing the number of BSN/MScN nursing hours by 10% (USD)
Pulmonary diseases	1126.0	2253.0	1	2715.0
Internal diseases	1468.7	1846.2	1	1705.5
Neurology	1660.5	1890.7	1	1545.0
Cardiology	1845.4	2161.3	1	1589.2
Total number of non-surgical units	6100.5	7630.4	4	1697.1

BNS, Bachelor of Nursing Science; MScN, Master of Nursing Science.

### 3.4 Sensitivity analysis

The aim of the analysis was to estimate the extent to which the planned intervention was at risk of inconsistency with the economic forecast. To this end, we changed the number of hours of BSN/MScN nurses, the cost of an hour of BSN/MScN nursing, the number of avoidable falls. A one-way sensitivity analysis was conducted with each parameter tested separately (Table 4).

**Table 4:** The parameter values used as the baseline for the sensitivity analysis and lower and upper values.

	Baseline	Difference value	Upper value	Lover value
The number of additional hours BSN/MScN	6100.9	305	6405.5	5795.5
The cost of one BSN/MScN nursing hour (USD)	9	0.1	9.1	0*
The number of falls	64	4	68	60
The number of avoidable falls	4.5	0.3	4.8	4.2

*No reduction in wages; **Standard deviation was 5%; BNS, Bachelor of Nursing Science; MScN, Master of Nursing Science; ***Standard deviation was 7%; for number of avoidable falls.

In the sensitivity analysis, the calculated value of the variable for a 10% increase in BSN/MScN nursing hours had a standard deviation of 5%, which was arbitrarily assigned to the parameters of additional BSN/MScN nursing hours and the cost per hour of BSN/MScN nursing care. For avoidable falls, the variable value was calculated at 7%, representing the standard deviation in the quarter with the highest number of falls.

**Figure 1:**
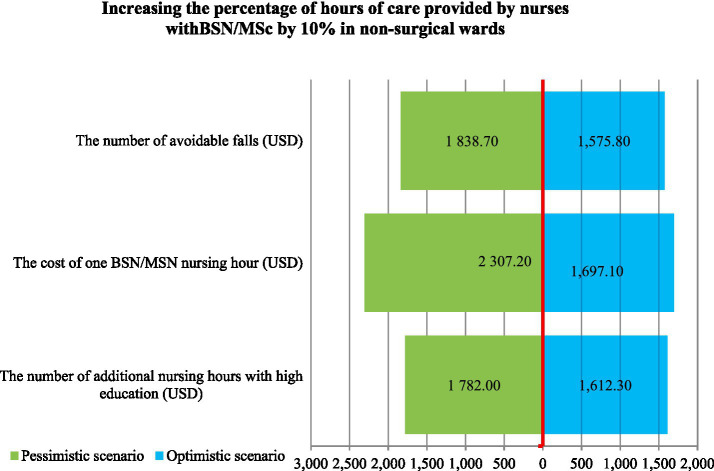
The sensivity analysis.

Simulation of the optimistic and pessimistic scenarios indicates the possible effects of a 10% increase in BSN/MScN nursing hours (Figure 1).

For the variable of BSN/MScN hours, we tested a 50.5% increase in BSN/MScN hours over the intervention, in comparison with the arbitrarily assigned increase by 5% (SD = 5%). In the pessimistic scenario, SD was subtracted from the number of BSN/MScN hours. In the optimistic scenario, SD was added to the baseline number of BSN/MScN hours.

In the pessimistic scenario for the “cost of 1 h of BSN/MScN” variable, arbitrarily assigned standard deviation SD = 5% was added to the cost of a BSN/MScN nursing hour. The analysis did not include a reduction in wages.

In the pessimistic scenario for the “avoidable falls in 2021” variable, we selected the quarter with the lowest number of falls. On this basis, the number of avoidable falls was calculated.

In the optimistic scenario, the quarter of 2021 with the highest number of falls was selected, and the number of avoidable falls was calculated. In both scenarios, SD = 7%.

The sensitivity analysis shows that with a 10% increase in the number of BSN/MScN nursing hours (6100.5 h), the cost of avoiding one fall is 1697.1 USD. In the optimistic scenario, the number of BSN/MScN nurses’ hours is reduced by 5% (to 5795.5 h) and CER drops to 1612.3 USD. In the pessimistic scenario, an increase in the number of hours of BSN/MScN nurses by 5% (up to 6405.5 h) results in an increase in CER = 1782.0 USD. In the pessimistic scenario, a 5% increase in the cost of a BSN/MScN hour (USD9.4) results in an increase in CER = 2307.2 USD. A reduced cost of a BSN/MScN hour was not analyzed.

In the optimistic scenario, if the number of falls increases to 68 (SD = 7%), CER decreases to 1575.8 USD and in a pessimistic scenario, the number of falls decreases to 60 (7%) and CER increases to 1838.7 USD.

According to the sensitivity analysis, the biggest threat to the cost-effectiveness of the 10% increase in the number of BSN/MScN nursing hours is the increase in the cost of BSN/MScN nurse hours. For the remaining parameters, the result does not change significantly, meaning it is not sensitive to the parameter.

From the provider’s perspective, the cost and benefit analysis demonstrated that increasing the number of BSN/MScN nurses by 10% is a cost-effective means of eliminating missed care resulting in falls. The cost–benefit ratio is 0.1. This means that every zloty invested in eliminating missed care (increasing nursing care) brings a profit of 0.1 PLN. The cost–benefit ratio is 14. This means that the benefit of every zloty invested in increasing nursing care is14 PLN (3.3 USD).

In every respect, the analysis indicates that the net benefit is positive (benefits outweigh costs), so the intervention should be implemented (Table 5).

**Table 5:** The cost–benefit analysis (CBA).

CBA parameters for avoidable falls for the intervention of 10% increase in BSN/MScN nursing hours		
CBA parameters	PLN	USD
The cost of increasing nurse staffing (along with basic salary)	5152755.4	1255208.3
Number of avoidable falls	4	4
Estimated value of nurses’ salaries in hospitalization costs	17659107.0	4301748.3
The value of the estimated cost of nurses’ salaries in the cost of hospitalization in terms of avoidable falls	70636428.0	17206993.3
Net monetary benefit	65483672.6	15951785.0
Cost–benefit ratio (CBR)	0.1	0.0
Benefit–cost ratio (BCR)	14.0	3.3

BNS, Bachelor of Nursing Science; MScN, Master of Nursing Science.

## 4 Discussion

The CEA we conducted evaluates the cost-effectiveness of a 10% increase in BSN/MScN hours to achieve the clinical effect of avoiding one fall The incremental cost of avoiding one fall (CER) by increasing the number of BSN/MScN nurses by 10% is USD 1697.1. The cost of additional BSN/MScN hours in non-surgical departments in 2021 it is USD 1.00 per hospitalized patient.

The study population included adult patients (7,305) from four specialized hospital departments in 2021. The intervention involved a 10% increase in the number of BSN/MSN hours compared to the standard level of BSN/MScN hours (40.5%). The intended health effect was to reduce the number of falls during hospitalization by eliminating missed care associated with insufficient BSN/MScN staffing. The cost-effectiveness analysis was carried out from the provider’s perspective, i.e., the hospital. We calculated the unit cost of an hour of nursing care and the cost of an hour of care provided by BSN/MScN nurses. The analysis involved the falls that occurred in 2021, and then we calculated avoidable falls.

In the next step, we calculated the unit cost of the effect the avoidable fall (CER). The analysis adopted two scenarios of data variability: pessimistic and optimistic. The sensitivity analysis model considered variables such as the number of hours of BSN/MScN care, the cost of hour BSN/MScN care, and the number of avoidable falls.

The results showed that the calculated cost of avoiding a single fall through the intervention was USD 1697.1, comparable to Cookson’s analysis (1907.5 USD = 1,412 GBP) (79). Similar results were obtained for the BSN/MScN hour cost variable, whose cost amounted to 2,307 USD—more than the increase in the number of BSN/MScN hours variable. In Cookson’s study, that cost was USD 3394.8 (GBP2513) and was > USD 1487.3. The results obtained for the “increase in the number of avoidable falls” variable were also close to Cookson’s results. In the presented study, CER was USD 1,576, compared to USD 1,634.6 (GBP 1,210) in Cookson’s study. A reduction in the number of falls was associated with an increase in CER = 1839 USD. In contrast, Cookson’s analysis indicated USD 2,410 (GBP 1,784).

The incidence rate for falls per 1,000 patient days estimated for the representative Polish population is significantly lower than in the Cookson study. The fall incidence rate was 1.5 per 1,000 patient days, and after a 10% increase in BSN/MScN hours, the incidence rate decreased by 1.4. The Incremental Change was 0.1. This means that four falls were prevented. In Cookson’s study, the fall incidence rate was higher before the intervention—14.62 per 1,000 patient days and higher after the intervention—12.22, in relation to nation-wide rates. The incremental change was 2.40. This meant that 22 falls were prevented. The difference in the number of falls that were prevented was 18. However, the average national fall rate per 100 patient-days in the United Kingdom, to which Cookson’s study referred, was 4.8 (80). According to Cookson, this indicates that in their own studies, the average hospitalization time is short and the risk is lower because it is measured only during hospitalization, which was 4.8 days.

Cookson’s research shows that increasing the percentage of BSN/MScN nurses above 60% results in fewer falls, and improving nurses’ education to reduce falls is cost-effective (79). In contrast, our own study proved that 50.5% is the percentage of graduate nurses which is cost-effective and results in a decrease in the number of falls.

The 10% increase in the number of hours proved to be both clinically effective and cost-effective in the conducted analysis. Each zloty (1 PLN) invested eliminating avoidable falls generates the benefit of PLN 14 PLN (3.3 USD).

There are no studies evaluating the cost-effectiveness of interventions for fall prevention in Poland. In the United States, “patient monitoring” intervention was studied from that perspective. It contributed to reducing the costs down to USD 5895 per fall (ICER) (16). Patient monitoring means eliminating missed care and, as a result, the risk of falls. The presented study operates on the assumption that the “increase in % of BSN/MScN nurses” intervention can also allow for more effective and efficient “monitoring of patients” and thus reduce the number of falls.

The cost of falls in Poland is also unknown. In the United States, the mean fall cost is estimated at USD 7,718 (79).

Poland has requirements concerning minimum employment standards, but the BSN/MSN level is not regulated (81).

Other fall prevention strategies are also researched, e.g., changing nursing staffing levels and assessing the cost-effectiveness of this intervention (45). ICER cost for direct fall care in the United Kingdom was estimated at USD 106.7 (79 GPD) per fall (45). In this study, the cost of effective prevention of one fall (CER) is USD 1,697.1. Thus, it can be concluded that this intervention can prevent generating costs associated with the treatment of complications and eliminate prolonged hospitalization, rehospitalization, rehabilitation and other permanent health effects for patients. Moreover, in Poland, the costs of care for post-fall patients are not separately monitored or shown in statistics as a separate cost of hospitalization. The costs of post-fall direct care and prolonged hospitalization are not specified.

The CEA analysis provides evidence that additional BSN/MScN hours contribute to eliminating avoidable falls. The intervention consisting in increasing care in order to eliminate missed care is cost-effective from both the provider Increasing the hours of nurses with higher education, based on a case–control study, reduces falls by 9%—the intervention is cost-effective. From the perspective of the hospital, in turn, the profitability depends on the profitability threshold established in the hospital. Staff salary is within 60% of the costs (82), and the cost of nurses is only 25.5–30.1% of the hospital budget per year (71). If we accept that nurses’ salaries are 25.5% of the total hospital budget, increasing the percentage of graduate nurses to 50.5% should be acceptable according to HB HTA. In addition, the salary increase due to a 10% increase in BSN/MScN hours is only 1 USD per patient (2021).

The level of nurses’ education plays a vital role in developing awareness and integrating professional values in practice (83). Nurse education is also associated with reduced mortality and rehospitalization of patients (84, 85). Also, knowledge validation through nursing certification has an impact on specific outcomes, such as patient falls and healthcare-related infections (86).

In compliance with the best-quality-to-price ratio procurement criterion, which is recommended in the EU for reimbursement of benefits (2), it is worth adding the criterion of 50.5% BSN/MScN to the criteria for ordering nursing care-related health services in Poland.

Due to the limited number of studies on the cost-effectiveness of fall prevention, further research should be expanded based on a larger population of patients and various hospital profiles to obtain more reliable results in the future.

### 4.1 Application in practice

The initiated studies could be used to design research in other hospitals and contribute to starting a discussion aimed at creating a national database of nursing-sensitive indicators to support healthcare policy development. The publication of the research results should lead to a proposal from nursing organizations to the Ministry of Health to include nursing-sensitive quality indicators in the executive regulations of the Quality Act and to design a data collection system for the discussed events through a platform containing medical records.

The study results could serve as a basis for discussions on how to finance healthcare services, which should be dependent on hospital care outcomes measured by quality indicators, as is the case in other countries, e.g., C-HOBIC (Standardized Information to Support Clinical Practice and Quality Patient Care), NQuIRE® (Nursing Quality Indicators for Reporting and Evaluation®) (54).

When managing databases of nursing-sensitive indicators, the quality and outcomes assessment model according to Donabedian (15, 87).

### 4.2 Limitations

The implementation of the intervention will depend on the willingness of the NHS to pay for the value for the patient (VBHC), which is the proven level of BSN/MScN in the study, which is necessary to eliminate missed care for falls. There is no access to data on the cost of falls (the cost of hospitalization for a femoral neck fracture from a hospital and social perspective), which could indicate the additional value of the proposed intervention. The presented results may be conducive to revising the AOTMiT (Polish Agency for Health Technology Assessment and Tariff System) service tariff, which will take into account the cost-effectiveness of BSN/MScN staffing. AOTMiT is expected to announce new estimates for all types of health services and update salaries in health care, including nurses’ salaries in 2024 (88). The lack of national records of adverse events in Poland prevents multicenter analyses, so this study is based on a single hospital. Another limitation is that not all costs were included in the analysis, the analysis only concerns interventions to increase care by nurses with higher education, other costs of the service provider were not counted.

## Data Availability

The datasets presented in this article are not readily available because the data are property of the hospital and have been made available to the researchers only for the purposes of the project. Resources contain non-anonymized data. Requests to access the datasets should be directed to beata.wieczorek-wojcik@apsl.edu.pl.

## Footnotes

^1^C34.2, C78.7, D38.6, D38.3.

^2^I63.0, I63.5, I64, I64.4, I63.3.

^3^A41.5, A41.8, A40.1.

^4^M51 0.1, G62.2, G98, G93.4, G40.3.

^5^J44.8, J96.1, J26.9, J96.0, J15.8.

^6^I29.9, I21.3, I25.0.

^7^K51.9, A04.7.

## Appendix

Appendix 1 A description of the clinical characteristics of the entire population of the study.

**Table:**

Departments	ICD 10—80 % main diagnosis general population (7,305 patients)	ICD 10—80% specific diagnosis population with falls (64 patients)
Non-surgical departments TOTAL	Heart failure I50 (9.47%),Atrial fibrillation and flutter I48 (7.96%),Acute myocardial infarction I21 (7.65%),Cerebral infarction I63 (7.15%), Chronic ischemic heart disease I25 (6.08%), Acute kidney failure N17 (4.97%), Other sepsis A41 (2.79%), Bacterial pneumonia J15 (2.26%), Malignat neoplasm of bronchia and lung.C34 (1.85%), Other interstitial pulmonary diseases J84 (1.54%), Other cardiac arrhythmias I49 (1.54%), Other disorders of nervous system, not elsewhere classified G98(1.53%), Neoplasm of uncertain or unknown behavior of middle ear and respiratory and intrathoracic organs D38 (1.51%), Atrioventricular and left bundle-branch block I44 (1.47%), Asthma J45 (1.44%), Other anemias D64 (1.42%), Congenital malformations of cardiac septa Q21 (1.39%), Other bacterial intestinal infections A04 (1.37%), Pulmonary eosinophilia, not elsewhere classified J82 (1.33%), Sleep disorders G47 (1.23%), Pulmonary embolism I26 (1.23%), Alcoholic liver disease K70 (1.18%), Angina pectoris I20 (1.09%), Other respiratory disorders J98 (0.99%), Urinary tract infection, site not specified N39 (0.98%), Sarcoidosis D86 (0.97%), Neoplasm of uncertain behavior of oral cavity and digestive organs D37 (0.91%), Nonrheumatic aortic valve disorders. I35 (0.90%), General examination and investigation of persons without symptoms and reported diagnosis Z00 (0.78%), transient cerebral ischemic attacks and related syndromes G45 (0.77%), Neoplasm of uncertain or unknown behavior of brain and central nervous system D43 (0.73%), Nontraumatic intracerebral hemorrhage I61 (0.70%), Gastritis and duodenitis K29 (0.66%), Deficiency of other nutrient elements E61 (0.62%), Respiratory failure, not elsewhere classified J96 (0.60%), Poisoning by primarily systemic and hematological agents, not elsewhere classified T45 (0.60%), Pneumonitis due to inhalation of food and vomit J69 (0.59%).	Cancer (14.1%) (C34.2, C78.7, D38.6, D38.3), Stroke (17.2%), (I63.0, I63.5, I64, I64.4, I63.3), Sepsis—6 (9.4%) (A41.5, A41.8, A40.1), Other disease nervous systems (12.5%) (M51.1, G62.2, G98, G93.4, G40.3), Other respiratory diseases (7.8%) (J44.8, J96.1, J26.9, J96.0, J15.8), Heart failure (3.1%) (N17.9), Heart attack (4.7%) (I29.9, I21.3, I25.0), Heart failure (3.1%), COVID 19 (4.7%) (U07.1), Gastrointestinal disease—(7.8%) (K51.9, A04.7), Kidney failure (6.2%) (N17.9).
